# Perforated gastric carcinomatosis following invasive lobular cancer of the breast

**DOI:** 10.1002/ccr3.2116

**Published:** 2019-04-10

**Authors:** Ivan De Gruttola, Md Tanveer Adil, Lorraine D’Souza, Periyathambi Jambulingam, Douglas Whitelaw

**Affiliations:** ^1^ Department of Upper GI and Bariatric Surgery Luton and Dunstable University Hospital Luton UK; ^2^ Department of Pathology Luton and Dunstable University Hospital Luton UK

**Keywords:** acute abdomen, breast cancer, gastric metastasis, total gastrectomy

## Abstract

Current guidelines do not advocate a routine search for gastric metastasis in known patients of breast cancer. This makes it challenging to suspect them clinically as they progress over many years. Gastric carcinomatosis from primary invasive lobular cancer can perforate leading to life‐threatening abdominal emergency necessitating surgery.

## 1 INTRODUCTION

Breast cancer is the most common malignancy and an important cause of cancer mortality in women.^1^ Distant metastasis rather than the primary tumor itself is the main cause of death.^2^ The distant organs to which breast cancer preferentially metastasizes are the bones, liver, lungs, soft tissue, adrenal glands and brain, in addition to lymph nodes. Gastrointestinal (GI) tract metastasis from a primary breast cancer is a rare phenomenon and lobular histology of the breast has the highest tendency to spread to the stomach, usually as linitis plastica.^3^, ^4^ Furthermore, gastric carcinomatosis is difficult to detect clinically due to the nonspecific symptoms and low detection rate on routine imaging used for staging of breast cancer.

Even though gastric metastasis and carcinomatosis from a breast primary have been occasionally described in literature, gastric perforation due to this dissemination is extremely rare. We present a rare case of gastric perforation secondary to gastric carcinomatosis treated with emergency total gastrectomy 8 years after primary diagnosis of invasive lobular carcinoma of the breast.

## 2 CASE HISTORY AND EXAMINATION

A 61‐year‐old female presented to the emergency with a 24‐hour history of abdominal pain associated with nausea and vomiting. There was no prior history of anorexia, abdominal discomfort, or hematemesis. She had a heart rate of 98 beats/min, respiratory rate of 19 per minute, blood pressure of 130/80 mm Hg, and a temperature of 37.5°C. Abdominal examination showed mild distension with tenderness and peritonism in the upper abdomen. Past history included an episode of supraventricular tachycardia that was treated with adenosine and a previously diagnosed grade 2 left breast infiltrating lobular carcinoma (ILC) treated with a skin‐sparing mastectomy and sentinel lymph node biopsy 8 years ago in 2010. The breast tumor was staged as pT3 (7 cm), N0, M0, estrogen receptor(ER) +ve, progesterone receptor(PR) +ve, and human epithelial growth factor receptor 2(*Her 2*) −ve. She had completed six cycles of adjuvant FEC 75 chemotherapy (epirubicin 75 mg/m^2^, 5‐fluorouracil 600 mg/m^2^, and cyclophosphamide 600 mg/m^2^) in 2010 followed by radiotherapy(40 Gy in 15 fractions) in 2011. She was started on tamoxifen in 2010 as she was intolerant to aromatase inhibitors due to gastrointestinal side effects and eventually discharged from the breast team in early 2018 after 8 years of follow‐up with an advice to continue tamoxifen for 2 more years.

## 3 INVESTIGATIONS AND TREATMENT

**Figure 1:**
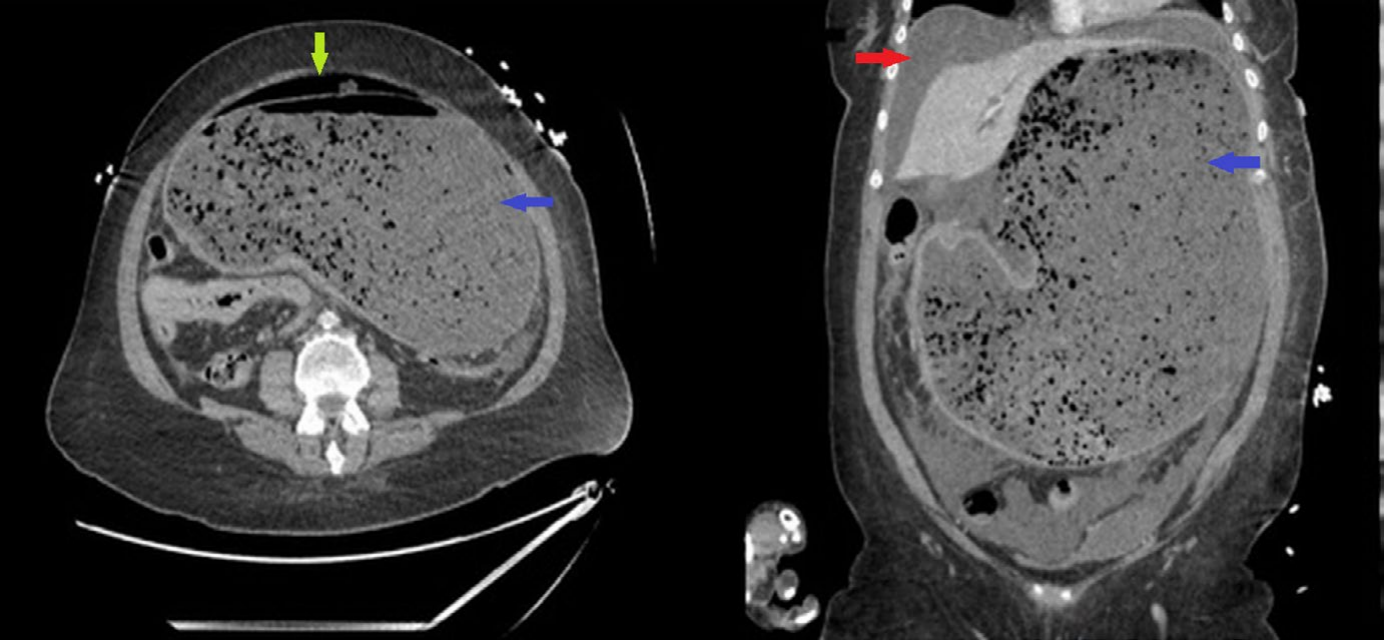
Computed tomography of the abdomen and pelvis (axial and coronal view) showing massive dilatation of the stomach containing food debris (blue arrow) with free intraperitoneal air (green arrow) and fluid (red arrow)

On admission, blood results showed a hemoglobin of 14.5 g/dL (normal range—12‐15 g/dL), white blood cell count of 13.1 × 10^9^/mm^3^ (normal range—4‐11 × 10^9^/mm^3^), C‐reactive protein of 2.4 mg/L (normal range—<0.6 mg/L), urea of 8.1 mmol/L (normal range—2.5‐7.1 mmol/L), sodium of 130 mmol/L (normal range—135‐145 mmol/L), potassium of 4.2 mmol/L (normal range—3.5‐5.0 mmol/L), creatinine of 109 μmol/L (normal range—50‐90 μmol/L), pH of 7.3 (normal range—7.35‐7.45), and base excess of −3 mEq (normal range—−2 to +2 mEq). An urgent contrast‐enhanced computed tomography (CT) of the abdomen and pelvis was performed that showed massive gastric dilatation associated with a large 7 cm sized perforation on the lesser curve of the stomach adjacent to the fundus and extraluminal discharge of gastric contents into the peritoneal cavity associated with free intraperitoneal gas and fluid in the peritoneum (Figure 1).

She was resuscitated with intravenous fluid, antibiotics, analgesics, and nasogastric suction. An emergency laparotomy was performed within 6 hours of admission. The entire peritoneal cavity was found to be full of fermenting solid and semisolid food. There was a large perforation on the lesser curve of stomach extending from the gastroesophageal junction to the incisura. The anterior wall and lesser curve of stomach were nonviable, and posterior gastric wall was of borderline viability. A total gastrectomy and Roux‐en‐Y esophagojejunostomy were performed along with copious peritoneal lavage with 15 L of normal saline. A jejunostomy tube was also inserted for feeding in the postoperative period. The surgery took 4 hours after which the patient was transferred to Level 3 care in a ventilated state. Jejunostomy feeding was begun in incremental quantities after 48 hours, and she was extubated after 72 hours. She was able to tolerate orally from the 13th postoperative day after which she was moved to ward‐based care. A follow‐up CT scan of the thorax, abdomen, and pelvis performed on the 15th postoperative day revealed bilateral pleural effusion that was treated with bilateral thoracentesis and sample was sent for study from the left side. The patient spent a further 2 weeks in the ward after which she was discharged in stable condition.

## 4 OUTCOME

**Figure 2:**
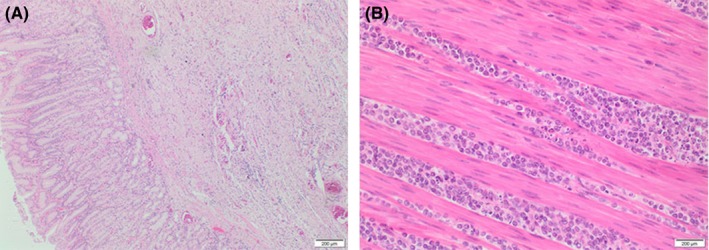
Histology of the stomach showing scattered metastatic lobular breast cancer cells on hematoxylin and eosin staining (A) and tumor cells infiltrating through the muscle fibers (B)

**Figure 3:**
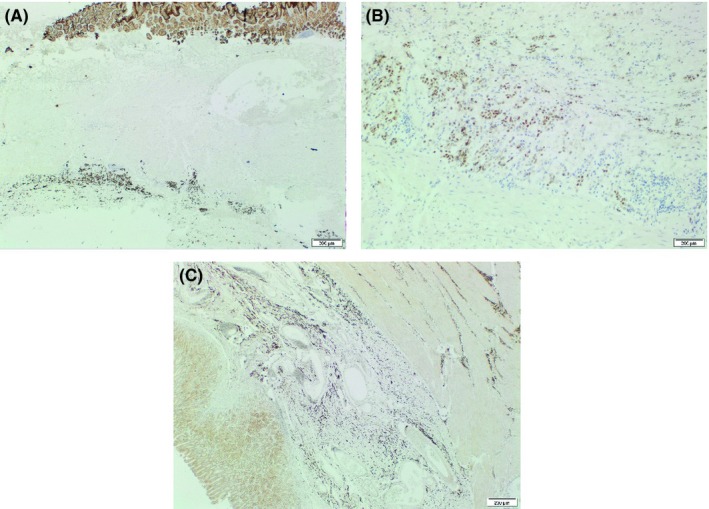
Immunohistochemistry of the stomach showing positivity for cytokeratin AE1/AE3 (A), diffuse moderate staining with estrogen receptor (B), and strong nuclear positive staining with GATA3 (C)

Stomach histology showed manifestations in keeping with metastatic origin rather than a primary gastric pathology. Groups, clusters, and cords of mildly atypical cells were seen in areas within the subserosal tissue (Figure 2). In other areas, atypical cells were seen infiltrating through the muscle into the submucosa (Figure 2). On immunohistochemical stains, these cells showed positive staining with cytokeratin (CK) AE 1/3 (Figure 3) and CK 7 along with diffuse moderate staining with estrogen receptor (Figure 3). *HER‐2* was negative (1+) showing a faint incomplete staining pattern. The cells did not express CK 20, synaptophysin, CD 56, chromogranin A, and TTF1. Furthermore, the tumor cells showed strong nuclear positive staining with GATA3 (Figure 3). This together with moderate ER staining was in keeping with a secondary cancer of the stomach from the breast.

Analysis of fluid obtained from left thoracentesis showed numerous neutrophilic polymorphs, scattered lymphocytes, and reactive mesothelial cells. Scattered among these were single and small groups of moderately atypical cells with abundant cytoplasm that were highlighted with GATA3 and CK 7 immunopositivity. ER and CK 20 were negative; however, in view of GATA3 positivity, a breast secondary was concluded.

She was started on exemestane after immunohistochemistry results and doing well at 6 months of follow‐up with no further episodes.

## 5 DISCUSSION

Breast cancer is the second most common cause of cancer death after lung cancer in developed countries.^7^ Almost 90% of breast cancer‐related deaths are due to tumor dissemination.^8^ A Japanese study reported a mortality rate of 11.6% in patients with metastatic breast cancer.^9^ Advanced invasive lobular carcinoma of the breast is shown to metastasize to bone (81%), lymph nodes (47%), lung (33%), liver (32%), peritoneum (30%), colon (26%), pleura (23%), adnexa (21%), stomach (16%), retroperitoneum (16%), and small bowel (11%) on staging CT scan of thorax, abdomen, and pelvis.^10^ A retrospective study conducted by Cummings *et al* on 197 autopsies performed on patients who died from metastatic breast cancer revealed that the 10.7% of the patients had metastasis in gastrointestinal tract.^5^ Stomach is an uncommon site for tumor metastasis, with an incidence of 0.2%‐0.7%.^11^, ^12^ In a large study on 1010 autopsies of patients who died from cancer, gastric metastasis was detected in 17 cases (1.7%) with the most frequent primaries being in the lung, breast, or melanoma.^13^

Metastasis to gastrointestinal tract can occur many years after the diagnosis of the primary cancer has been made.^13^, ^14^ According to a study, gastric metastasis is detected at an average of 75.6 months after a diagnosis of primary breast cancer has been made.^15^ This long interval period between primary cancer and gastric metastasis has also been reported for other malignancies like prostate cancer (9 years) and renal cancer (20 years).^16^, ^17^

Invasive ductal carcinoma (IDC) and invasive lobular carcinoma (ILC) of the breast have been shown to have differing metastatic patterns.^18^ IDC is the most common breast primary accounting for 75% of cases, while ILC accounts for 5%‐10%.^19^ IDC follows the more conventional metastatic pattern known in breast cancers, while ILC frequently involves unusual metastatic sites such as the GI tract and meninges.^20^ According to a Canadian study on 74 ILCs of breast with metastasis, atypical sites were involved in 41.9% with gastric metastasis occurring in 6.7%.^21^ This atypical pattern of metastasis in ILCs compared to IDCs can be explained by virtue of loss of expression of epithelial‐cadherin in ILCs.^22^

Symptoms of gastric metastasis include anorexia, abdominal pain, pyrosis, vomiting, and dysphagia making it difficult to distinguish gastric metastasis from primary gastric cancer.^16^ This case highlights the difficulty in differentiating gastric carcinomatosis from primary gastric cancer in an acute setting, where the diagnosis was confirmed after immunohistochemistry. Furthermore, this is the first described case of gastric carcinomatosis from breast cancer treated with total gastrectomy, highlighting the importance of maintaining a high index of suspicion for gastric metastasis in patients with a history of breast cancer who present with acute abdomen, despite being on hormonal treatment. Current guidelines for breast cancer do not include a site‐specific search for gastrointestinal metastasis either before surgery or at follow‐up in the form of endoscopic surveillance. Moreover, lack of specific symptoms should alert clinicians to focus on new diagnostic tools in the management of breast cancer.

## CONFLICT OF INTEREST

None declared.

## AUTHOR CONTRIBUTION

IDG: collected the data and drafted the manuscript. MTA: finalized the manuscript and involved in the management of the patient and corresponding author. LD: evaluated the histopathology slides and provided valuable contribution on immunohistochemistry of the specimen. PJ and DW: provided valuable guidance toward preparation of the manuscript and involved in the management of the patient.
